# The effect of socioeconomic status on the severity of behavioral and psychological symptoms of dementia and ADRD caregiver outcomes: a preliminary exploration

**DOI:** 10.1002/alz70858_104864

**Published:** 2025-12-25

**Authors:** Jordan R. Hill, Miriam J. Rodriguez, Bailey Gardner, Mariana D. Stavig, Lilian Golzarri‐Arroyo, Anna L. M. Macagno, Roger S. Zoh, Richard Holden, Malaz Boustani

**Affiliations:** ^1^ Indiana University School of Public Health, Bloomington, IN, USA; ^2^ Indiana University Center for Aging Research, Regenstrief Institute, Indianapolis, IN, USA; ^3^ Indiana University School of Medicine, Indianapolis, IN, USA; ^4^ Regenstrief Institute, Indianapolis, IN, USA; ^5^ Indiana Clinical Translational Science Institute, Indianapolis, IN, USA

## Abstract

**Background:**

Socioeconomic status (SES) is associated with health disparities across populations, including older adults with Alzheimer's disease and related dementias (AD/ADRD) and their caregivers. How SES affects the severity of behavioral and psychological symptoms of dementia (BPSD), burden, social support, and depressive symptoms among caregivers is underexplored. The aim of the current study was to investigate the relationship between SES and these outcomes.

**Method:**

Using baseline data collected from a larger study (*N* = 144), an ANCOVA was performed to compare BPSD severity, caregiver burden, depressive symptoms, and perceived social support between SES groups (race, education level, household income, employment, and insurance status).

**Result:**

The ANCOVA demonstrated no significant overall differences in BPSD severity related to SES measures. Pairwise comparisons revealed caregivers employed full‐time reported significantly lower BPSD severity than retired caregivers. Additionally, caregivers who were insured under Medicare plus another form of insurance (e.g. Medicare Advantage) reported significantly lower BPSD severity than caregivers with private insurance.

There was a significant difference in caregiver perceived social support between income levels, with caregivers having a household income greater than $75,000 reporting higher social support than those earning less than $75,000. While the ANCOVA comparing burden and depressive symptoms to SES demonstrated no statistical significance, pairwise comparisons demonstrated a) caregivers who attained trade school or associate degrees or attended some undergraduate education reported higher caregiver burden than those who had high school degrees or lower, b) employed caregivers (full‐ and part‐time) had lower burden than unemployed caregivers, c) caregivers with graduate degrees had less severe depressive symptoms than those with bachelor's degrees, and d) caregivers employed full‐time had less severe depressive symptoms than unemployed caregivers.

**Conclusion:**

Significant differences in BPSD severity and caregiver outcomes were identified between SES groups. Some of these differences reinforce previous findings (e.g. unemployment resulting in higher burden) while others added to the literature (e.g. caregivers employed full‐time reporting lower BPSD severity). The effects of SES on BPSD severity and caregiver burden are important to consider when creating interventions. Future studies should focus on exploring these relationships with larger samples and with other groups (e.g. Hispanic/Latino/a ethnicity, other racial identities).

